# The evolution of testicular sperm extraction and preservation techniques

**DOI:** 10.12703/b/9-2

**Published:** 2020-10-30

**Authors:** Erica S Godart, Paul J Turek

**Affiliations:** 1 The Turek Clinic, 55 Francisco Street, Suite 705, San Francisco, CA 94133, USA

**Keywords:** Azoospermia, sperm retrieval, sperm, testicular sperm, testis failure, TESA, TESE, sperm mapping, fine needle aspiration, hypogonadism, testosterone, IVF-ICSI

## Abstract

Along with the advent of intracytoplasmic sperm injection in 1992, sperm retrieval procedures now allow the possibility of conception from male sterility. In cases of sterility due to blockages in the reproductive tract, sperm retrieval procedures are relatively straightforward and reliable. In nonobstructive azoospermia or testis failure, sperm often can be difficult to retrieve. For this reason, the field of testicular sperm retrieval has witnessed tremendous change and innovation to achieve higher sperm yields, increasing efficiency and safety, along with fewer complications. We review the history and evolution of testicular sperm retrieval since its inception. Using the findings from randomized controlled trials, basic science studies, meta-analyses, case-controlled or cohort studies, best-practice policies, and literature reviews, we outline the concepts, facts, and principles that have been elucidated over several decades of experience with sperm retrieval. We also appraise the merits and issues of the most popular sperm retrieval techniques and strategies. Finally, we define areas of future clinical and laboratory development that will further refine the field of testicular sperm retrieval.

## Introduction

Testicular sperm retrieval is now almost 30 years old^1^. For the first time in history, it has allowed men who lack ejaculated sperm because of either testis failure or unreconstructable blockages the opportunity to become fathers. So, what have we learned? We begin our discussion of testicular sperm extraction (TESE) procedures by reviewing the evidence-based principles that have surfaced since its inception.

The first thing is to define the playing field. Azoospermia, which is the lack of sperm in the ejaculate, is either obstructive or nonobstructive^2^. Obstructive azoospermia (OA) results from acquired or congenital conditions that block the passage of sperm from the testicle through the reproductive tract. Among these conditions are infections, idiopathic causes, ejaculatory duct obstruction, prior vasectomy, and the congenital absence of the vas deferens. Nonobstructive azoospermia (NOA) is due to testicular failure and impaired production of mature sperm. Among the common primary causes are infection, torsion, cryptorchidism, chemotherapy, and Y-chromosome microdeletions or karyotype abnormalities. Secondary causes, such as prolactinoma and Kallmann syndrome, result from faulty pituitary and hypothalamic signaling and are often hormonally correctable.

The first principle learned from testicular sperm retrieval is that it is typically more difficult to perform in men with NOA than in those with OA. This is largely because spermatogenesis in men with NOA can be “patchy”, occurring in “islands” unlike the uniformly and globally normal sperm production in men with obstruction^3,4^.

**Figure 1.**
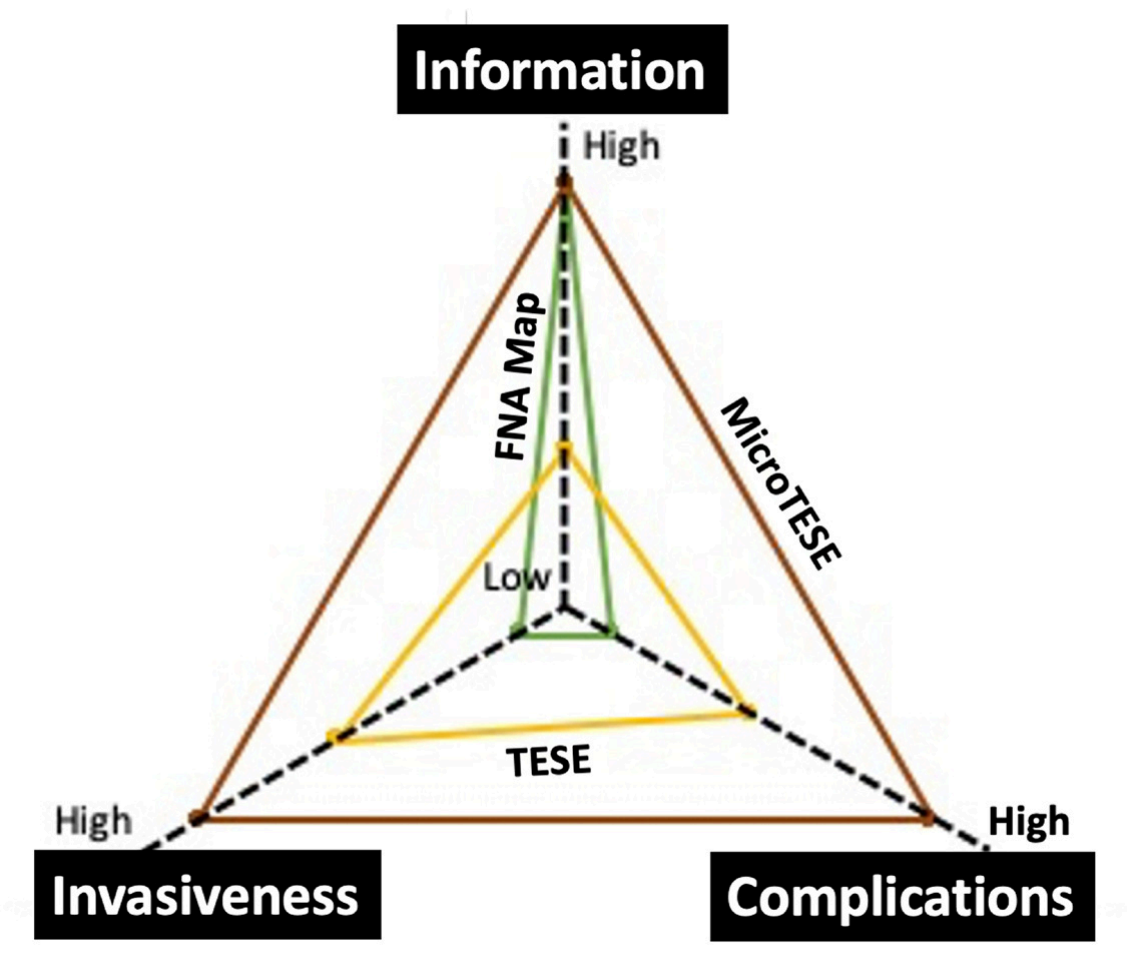
Radar plot comparing the qualities of three sperm retrieval techniques based on information obtained, invasiveness and complications. FNA Mapping (green line), TESE (yellow line), MicroTESE (brown line). FNA, fine needle aspiration; MicroTESE, microdissection testis sperm extraction; TESE, testis sperm extraction.

A second concept we realized is that testicular sperm retrieval procedures not only can fail but also can do permanent damage to the testicle and lead to hypogonadism or lower testosterone levels^5,6^. Given that many couples might need multiple *in vitro* fertilization-intracytoplasmic sperm injection (IVF-ICSI) cycles and sperm retrievals for a successful conception, there is an onus on clinicians to develop (a) efficient sperm retrieval techniques that, (b) maximize yield, (c) minimize procedure extent and morbidity, and (d) allow sperm cryopreservation to avoid repeated procedures (Figure 1). A Cochrane review of the literature on techniques of sperm retrieval found a lack of randomized controlled trials on which to base a recommendation for one sperm retrieval technique over another^7^. The only hard and fast recommendation was to select the least invasive and simplest technique for sperm retrieval whenever possible. Surely reproductive urologists, like other physicians, should be guided by the Hippocratic oath to “First, do no harm”. Indeed, this had led to several creative strategies that seek to optimize the safety and success of surgical sperm retrieval.

Lastly, it has also become clear that the performance characteristics of testicular sperm in IVF-ICSI obtained from men with NOA is inferior to those from men with OA^8^. This finding is independent of the type of sperm retrieval procedure used^7^, which suggests that it may reflect the impact of the genetic etiologies that underlie a higher proportion of NOA than OA men who want to have children.

## Testicular sperm retrieval in azoospermia

Since 1993, TESE has been routinely employed in men with OA and essential for men with testicular failure or NOA^1^. For men with OA, procedures to retrieve testicular sperm generally can be performed under local anesthesia in an office setting. The procedures needed to extract sperm from men with NOA are considerably more complicated^9^.

### Obstructive azoospermia

OA results from blockage in the reproductive tract. By definition, sperm production is quantitatively normal. The most common sperm retrieval method for patients with OA is needle aspiration (testicular sperm aspiration, or TESA) or by percutaneous biopsy or open surgical biopsy (TESE). We favor the TESA procedure in which an angiocatheter is inserted percutaneously into the testis and the needle is withdrawn, leaving the soft catheter in place^2^. After the application of 20 mL of negative suction to the catheter through arterial tubing, testis tissue can be atraumatically drawn into the catheter and tubing and expelled into medium for processing. Typically, sufficient sperm are retrieved such that they can be either timed with oocyte harvest or performed in advance and cryopreserved for future use. The risk associated with TESA procedures is minimal. Using a biopty gun-TESE procedure results in hematoma in 1 to 5% of cases as assessed by ultrasound. Open TESE procedures have similar risk, consisting mainly of bruising and bleeding (<5%). With repeated testicular sperm retrievals in patients with OA, there is certainly the risk of procedure-induced hypogonadism as Leydig cells are removed indiscriminately with sperm-containing seminiferous tubules.

### Nonobstructive azoospermia

Men with diagnosed NOA produce reduced or no levels of mature sperm because of testicular failure. In addition to being low, sperm production in NOA testes is typically “focal” or “patchy” in nature, making simple TESA procedures less effective than TESE for successful sperm retrieval. Indeed, over the last two decades, there has been enormous progress in developing more targeted, efficient, safer, and less invasive methods of sperm retrieval in men with NOA. The various strategies that have been developed are reviewed here. Notably, even after several decades in which these techniques have been in use, there is no level-1 evidence to support one sperm retrieval method over another.

***Multibiopsy testicular sperm extraction.*** One of the earliest strategies described to improve the chance of finding sperm in men with NOA was the multibiopsy TESE technique^10^. Developed in 1997, it was based on the concept that increasing sample size will increase the likelihood of finding sperm. Compared with a single testicular incision in simple TESE procedures, multibiopsy TESE involves multiple testicular incisions (up to 15) in different regions of the organ with tissue extraction until sufficient sperm are obtained. The original description of the technique in 21 patients found sperm in 70% of patients^10^. A more recent study (n = 741 patients) using the multibiopsy approach found that sperm retrieval rates ranged from 44% for a single biopsy to 58% for four biopsies^11^. Other contemporary studies of hundreds of men demonstrate sperm retrieval rates of 47 to 48% using a multibiopsy approach^12,13^. However, no randomized prospective studies have directly compared TESE and multibiopsy TESE techniques for either efficacy or safety.

**Figure 2.**
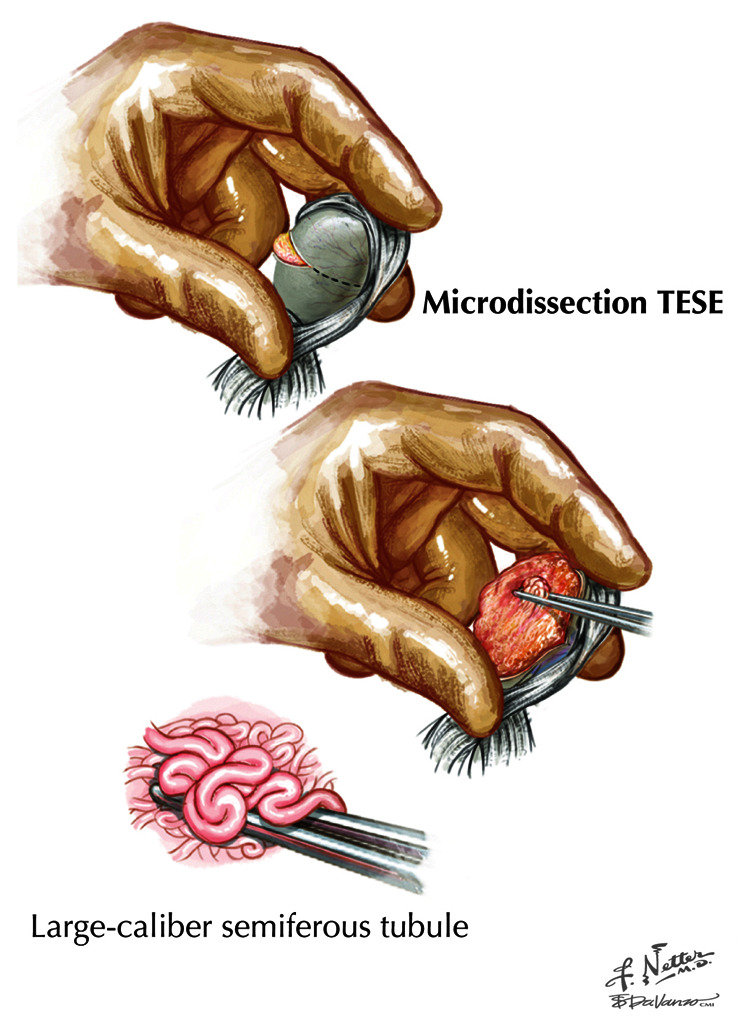
Illustration of the technique of microdissection testis sperm extraction (TESE) for sperm retrieval. A single large equatorial or longitudinal incision is used to access the entire testis parenchyma and larger and more opaque seminiferous tubules selected for biopsy with the help of operative microscopy. Reprinted with permission from Elsevier Press^21^.

***Microdissection testicular sperm extraction.*** Soon after the description of multibiopsy TESE, Schlegel *et al*. (1998) developed microdissection TESE that allowed improved localization of testicular sperm with the aid of intraoperative microscopy^14^. Microdissection TESE developed from observing that seminiferous tubules with active spermatogenesis seem bigger and more opaque under magnification than those with inactive production. In terms of procedure, the testicle is exposed in a fashion similar to that of a standard TESE. But a much larger longitudinal or equatorial incision is made through the tunica albuginea over the full length or width of the testicle, and the testicular parenchyma is wholly extruded through this incision (Figure 2). The exposed seminiferous tubules are studied by operative microscopy and are selectively biopsied for sperm. Systematic reviews comparing microdissection TESE with simple or multibiopsy TESE demonstrate an absolute sperm retrieval rate advantage of about 15 to 20% with microdissection^15,16^. Further analyses suggest that microdissection TESE is not superior to conventional TESE in cases of maturation arrest when tubules are of uniform size which reduces optical discrimination^6,17,18^ but performs better than conventional TESE with Sertoli cell–only histology, where sperm-laden tubules are more easily differentiated from surrounding sperm–free tubules^19^. Notably, no randomized controlled trials have confirmed the superiority of the microdissection TESE technique for sperm retrieval.

**Figure 3.**
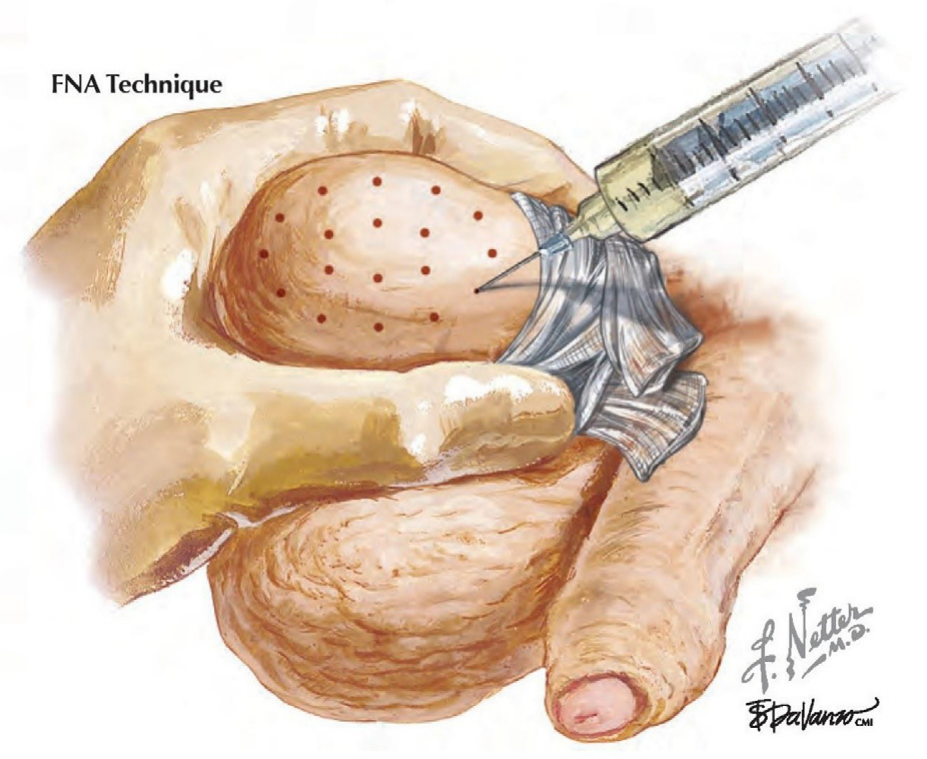
Illustration of the fine needle aspiration (FNA) mapping technique. Cytologic samples are taken from a grid-like, templated pattern from the testis. Reprinted with permission from Elsevier Press^21^.

***Fine needle aspiration mapping and map-directed testicular sperm extraction.*** Another popular strategy to find sperm in NOA men takes a completely different conceptual approach. Similar to the concept of using GPS to plan your travel by car, testicular fine needle aspiration (FNA) “mapping” is a non-surgical diagnostic procedure that identifies pockets of sperm in the NOA testis^4^. A series of 18 testicular aspiration samples are taken in a templated manner encompassing the entire surface and depth of the testicle (Figure 3). Each aspirated specimen is pap-stained and read cytologically for the presence or absence of mature sperm and also for all classic histologic patterns. A subsequent surgical sperm retrieval is planned with and guided by the location and presence of sperm on the map^20^. Ultimately, by “knowing before you go” with mapping, sperm retrieval procedures are shorter, more focused, less extensive, and more likely to find sperm than otherwise “blind” TESE procedures^2^.

**Figure 4.**
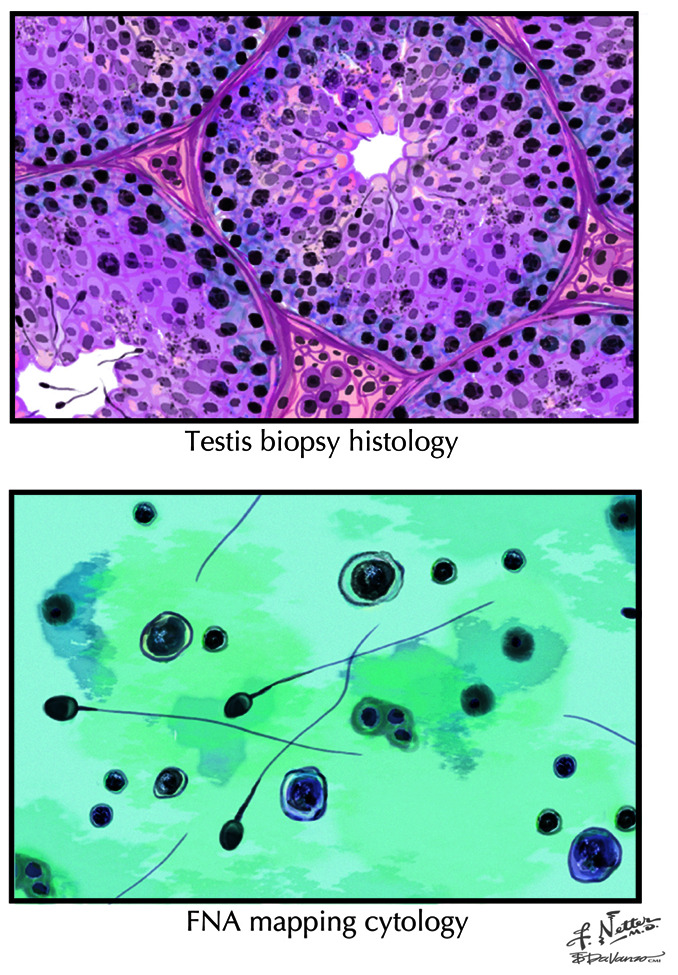
Illustrations of the findings obtained from a testis biopsy (upper panel) and testis cytology (lower panel). Given the differences in cell density between these procedures, it is much easier to identify mature sperm on cytology. FNA, fine needle aspiration. Reprinted with permission from Elsevier Press^21^.

**Figure 5.**
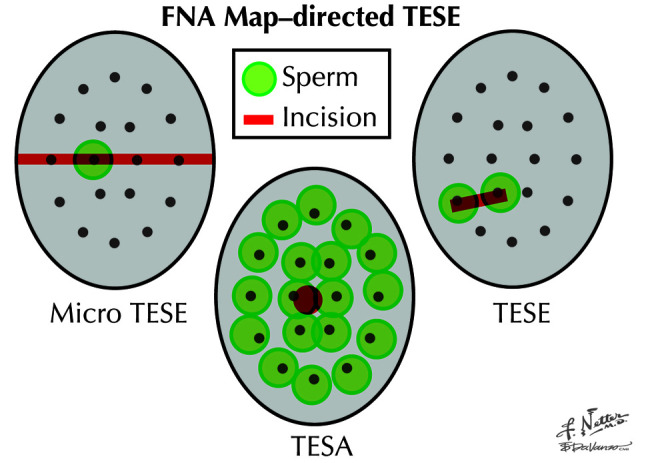
Approach to sperm retrieval after fine needle aspiration (FNA) mapping. Green circles represent areas of the testicle with sperm. Red lines and circles are planned incision or aspiration sites. After FNA mapping, sperm retrieval can be targeted and simplified when compared with otherwise “blind” testis sperm extraction (TESE) procedures. TESA, testicular sperm aspiration. Reprinted with permission from Elsevier Press^21^.

Thus, the essential differences between the FNA mapping-directed TESE approach and traditional TESE procedures are the following: (a) It is a “liquid biopsy” technique that paints a picture of sperm presence, location, and density within the testicle before a sperm retrieval is attempted; (b) it employs the precision of diagnostic cytology to identify sperm and does not rely on qualitative measures such as seminiferous tubule size to find sperm (Figure 4); and (c) by knowing exactly where sperm are located in the testicle beforehand, it focuses sperm retrieval procedures to one side or another and to specific areas within the testicle, thereby minimizing procedure time, complexity, and extent along with increasing sperm yield (Figure 5)^2^.

To illustrate these principles, we recently reported on the patterns of sperm found with FNA mapping in 82 men in whom prior bilateral microdissection TESE procedures failed to find sperm^22^. Overall, mature sperm were found in 29% of men by FNA mapping after failed microdissection procedures performed elsewhere. Moreover, sperm digital “heat maps” showed that sperm were found mainly in the testis periphery and not centrally, suggesting that microdissection procedures are biased toward central sampling of the testicle. In addition, when follow-up sperm retrieval procedures guided by FNA mapping were performed, sufficient sperm were retrieved for all eggs at IVF in 100% of attempts and surplus sperm were cryopreserved in 67% of cases. Notably, unilateral procedures were sufficient for sperm recovery in 87% of cases (bilateral in 13%). Lastly, sperm were found in completely undilated (36% of cases) or only marginally dilated (31% of cases) seminiferous tubules, which suggests that preferential biopsy of enlarged and opaque seminiferous tubules, the *sine qua non* for finding sperm with microdissection TESE, is neither necessary nor sufficient for successful sperm retrieval in NOA.

## Outcomes of testicular sperm retrieval

### Outcomes using nonobstructive azoospermia versus obstructive azoospermia sperm

How does the clinical performance of testicular sperm from men with OA differ from that of men with NOA with IVF-ICSI? A meta-analysis of available non-randomized data comparing ICSI results (n** = 1103 cycles) from men with NOA versus OA found significantly worse outcomes in cases of NOA^8^. In a fixed-effects analysis, OA cases were associated with significantly higher normal fertilization rates (relative risk [RR] 1.18, 95% confidence interval [CI] 1.13–1.23) and clinical pregnancy rates (RR 1.36, 95% CI 1.10–1.69) when compared with NOA cases. A non-significant increase in ongoing pregnancy rates was also detected (RR 1.19, 95% CI 0.87–1.61). However, no differences in implantation rates (RR 1.01, 95% CI 0.87–1.61) or miscarriage rates (RR 0.84, 95% CI 0.48–1.48) were observed. This may be true as a significant proportion of men with NOA, unlike those with OA, harbor underlying genetic conditions that might impact embryo and fetal development.

### Outcomes of fresh versus frozen-thawed testicular sperm

At its inception, testicular sperm retrieval was performed concurrently with egg retrieval at IVF-ICSI to provide “fresh” sperm. To simplify the complex logistics of timing fresh sperm retrieval to egg retrieval, some centers began to perform testicular sperm retrievals in advance of egg retrieval and freezing sperm for later thaw and use. From this experience came the observation that frozen-thawed testicular sperm showed a significant decrease in the proportion of motile sperm compared with fresh sperm. This observation was significant because IVF labs typically rely on sperm motility to choose a viable sperm for ICSI and having fewer or no motile sperm after thawing might mean that eggs would be injected with non-viable sperm.

Sharing this concern that frozen-thaw testicular sperm may not be as viable as fresh sperm for IVF-ICSI, we studied the cryobiological behavior of sperm from various anatomic sites within the reproductive tract^23^. Examining the effects of cryopreservation on the viability and motility of sperm harvested from the vas deferens, epididymis, and testis, we made several fundamental observations. First, regardless of anatomic source, all mature sperm tolerate the freeze–thaw process similarly in that about half of the initial population of viable sperm survives after thaw. Second, unlike the sperm viability findings, the recovery of sperm motility after thaw varies widely by anatomic source. In general, vasal sperm has better recovery of motility than epididymal sperm, which has better recovery than testicular sperm. The mean viability (by vital stain) and motility of fresh testicular sperm were assessed at 86% and 5%, respectively, whereas those of frozen-thawed testicular sperm were 46% and 0.2%, respectively. So, although there is a 50% recovery of viable sperm after thaw, only 4% of initially motile sperm are recovered after thaw. We concluded that the high viability of fresh testicular sperm, despite having a very low motility, makes either motile or non-motile fresh sperm functionally equivalent for ICSI. However, the significant decrease in sperm motility and viability observed with frozen-thawed testicular sperm could result in lower IVF-ICSI success. These findings argued in favor of continuing to use fresh testicular sperm for IVF-ICSI.

Subsequently, a meta-analysis that examined the impact of using fresh versus frozen-thawed sperm on actual IVF-ICSI success rates was published^8^. Examination of 1476 cycles and 1377 transfers involving fresh and frozen-thawed testis sperm from both patients with NOA and those with OA suggested that there was no difference in fertilization rates and ongoing pregnancy rates but that fresh testicular sperm was associated with a significantly higher implantation rate (RR 1.32, 95% CI 1.02–1.71; *P* = 0.04) and a borderline significantly higher clinical pregnancy rate (RR 1.16, 95% CI 1.0–1.35; *P* = 0.06) when compared with frozen-thawed testicular sperm. A more recent review and meta-analysis of 17 studies involving 1261 ICSI cycles in patients with only NOA revealed no differences in implantation rate (RR 0.93, 95% CI 0.66–1.30) or clinical pregnancy rate (RR 1.03, 95% CI 0.86–1.24) outcomes when fresh versus frozen-thawed testicular sperm were compared^24^. In addition to viability differences between fresh and frozen-thawed testicular sperm, observed increases in sperm DNA fragmentation rates after thawing of testicular sperm might also explain any differences in clinical outcomes^25^.

As the debate continues about whether frozen-thawed testicular sperm is clinically equivalent to fresh sperm, creative approaches regarding the timing of fresh testicular sperm retrieval have ensued. Studies of *in vitro* testicular sperm motility over time have shown that, in cases of both NOA and OA, sperm motility increases over 24 to 48 hours^26,27^ and the effects of incubation time on testicular sperm DNA integrity are modest and well described^25^. Performing a “fresh” sperm retrieval 24 to 48 hours in advance of egg retrieval offers considerable scheduling flexibility yet without significant deterioration in sperm clinical performance at ICSI.

### Hypogonadism after testicular sperm retrieval

As the invasiveness of surgical procedures used to find testicular sperm in NOA has increased, so have safety concerns, specifically those of surgically induced hypogonadism or testicular failure. In fact, it is remarkable how little study has been dedicated to this issue over the last several decades given its large and durable effect on patient health and overall quality of life. It is generally believed that the smaller the TESE samples taken, the less chance of postoperative hypogonadism^19^. With a single albeit large incision and more precise tissue dissection, microdissection TESE was initially thought to be less invasive than simple TESE procedures. However, with time and wider surgical experience, it appears that serum testosterone levels recover to baseline in only 50 to 90% of patients 1 year after microdissection TESE in experienced hands and with adequate clinical follow-up^6,28–31^.

A recent systematic review and meta-analysis that reviewed 15 non-randomized, retrospective, uncontrolled studies of testosterone levels before and after TESE procedures shed more light on this issue^32^. Among men with OA and NOA having TESE procedures (n = 12 studies), a statistically significant decrease in testosterone levels occurred for up to 12 months after the procedure and might put patients at risk of “temporary hypogonadism”. The degree of impairment was most marked in men with Klinefelter syndrome. In addition, a full recovery of testosterone levels was noted at 18 months among study patients. Significant limitations of this analysis include a wide heterogeneity in procedures performed (that is, TESA, TESE, and microdissection TESE), the inclusion of OA and NOA patient populations that may have different risk profiles for hypogonadism, and the generally poor quality of patient follow-up among included studies.

We have analyzed long-term changes in testosterone levels in an otherwise healthy, eugonadal population of NOA men referred to us after having had bilateral microdissection TESE procedures that failed. In essence, this study cohorts represents a highly select cohort of patients who had the “lowest risk” of hypogonadism before surgical sperm retrieval and who then underwent the most extensive sperm retrieval procedures (that is, procedures that failed to find sperm). We noted that, at a mean of 19 months after microdissection TESE (n = 35 men), serum total testosterone levels were 88 ng/dL lower than preoperatively. In addition, 30% of these low-risk, eugonadal men became clinically hypogonadal (<300 ng/dL) after microdissection TESE. We conclude that microdissection TESE procedures impart a significant risk of hypogonadism, even among healthy, eugonadal patients.

Overall, the current literature suggests that there is a significant risk of temporary and even permanent hypogonadism after TESE procedures. Whether the elevated risk of hypogonadism is related to the number or size of testicular tissue samples excised, the number and size of the testicular tunical albugineal incisions, or the surgical skill needed to perform sperm retrievals is entirely unclear. Nevertheless, the elevated risk of post-TESE hypogonadism has led some to advocate a “stepwise” approach to finding sperm in which a single TESE sample is taken, followed by a microdissection TESE using the same testicular incision, followed by a multibiopsy approach on the opposite testis if needed^33^. Others now perform only unilateral microdissection TESE procedures at the time of sperm retrieval. We believe that sperm FNA mapping is another minimally invasive pathway with the potential to be more “testis-preserving” in that (a) NOA men showing no sperm on FNA mapping are advised against surgical sperm retrieval altogether, thus avoiding surgical procedures entirely; (b) NOA men with FNA maps showing sperm may need only TESA or simple TESE procedures for surgical sperm retrieval, reducing the need for larger and more invasive procedures^20^; and (c) NOA men with FNA maps showing sperm are far more likely (80–85%) to need only unilateral procedures to retrieve sufficient sperm, thus sparing contralateral testicles from surgery^22^.

## Testicular sperm identification and cryopreservation

There is uniform agreement in andrology that improved laboratory identification and sorting of testicular sperm as well as better cryopreservation techniques offering improved sperm recovery are urgently needed. These goals align well with the concept (outlined earlier) of performing the most efficient and safest procedures possible, and performing them only once, in a population of men at high risk of hypogonadism. With sperm identification, it is clear that laboratory effort matters enormously to sperm retrieval success^34^. In our center, the laboratory “sperm search” time allotted for sperm identification during sperm retrievals varies with the procedure performed and ranges from 1 to 2 hours for TESA/TESE procedures to 4 to 6 hours for microdissection TESE procedures^2^. Additionally, testis tissue processing and digestion techniques that can increase sperm yield have been described^35^. Finally, the potential of microfluidic technology to aid in sorting mature sperm from testicular tissue appears promising^36^.

Improving the yield of viable testicular sperm after cryopreservation will also help to reduce the need for repeat procedures in men with NOA. Sperm freezing technology is now over 50 years old and its safety and efficacy are well known. However, the need to improve the yield of viable testicular sperm for ICSI after cryopreservation and thawing is more acute than ever since many men with NOA have low reservoirs of testicular sperm that are entirely depleted after a single sperm retrieval procedure. The application of sperm vitrification, an alternative to the traditional, computer-controlled slow-freezing process that is now commonly performed with oocyte freezing, has met with significant success^37^. Recent research has also addressed the value of *in situ* whole tissue cryopreservation rather than dispersed tissue freezing to improving the recovery of testicular sperm^27^. Innovative ways to find viable testicular sperm in the setting of an entirely immotile population after sperm thawing using motility stimulants, hypoosmotic swelling, or laser technologies have also been explored^38^. Lastly, novel freezing technology based on the excellent freezing characteristics of the zona pellucida offers the promise of improving sperm viability after freezing and thawing^39^. We believe that advances in laboratory technology over the next several years will have a major impact on our ability to help men with NOA to become biological fathers.

## Conclusions

Testicular sperm retrieval procedures have undergone an impressive evolution over the past several decades. Improvements in surgical technique and evidence-based and codified protocols in the laboratory handling of tissue have led to increased sperm yields. However, the best evidence is still insufficient to recommend any one sperm retrieval strategy over another for NOA as each has its strengths and limitations. One concern, however, is the relatively consistent finding of some degree of surgically induced hypogonadism after testicular sperm retrieval procedures, an issue with lifelong impact on affected patients. Guided by the principle of minimizing the number of procedures performed on azoospermic men in the setting of inefficient IVF outcomes, investigators have made progress in developing more efficient, less invasive, and safer sperm retrieval procedures, advances in sperm identification in the laboratory, and applying novel technologies to increase the yield of viable sperm after freezing and thawing.
